# Loss of PDCD4 contributes to enhanced chemoresistance in Glioblastoma Multiforme through de-repression of Bcl-xL translation

**DOI:** 10.18632/oncotarget.1154

**Published:** 2013-07-28

**Authors:** Urszula Liwak, Lindsay E. Jordan, Sally Davidson Von-Holt, Poonam Singh, Jennifer E.L. Hanson, Ian A. Lorimer, Federico Roncaroli, Martin Holcik

**Affiliations:** ^1^ Apoptosis Research Centre, Children's Hospital of Eastern Ontario Research Institute, Ottawa, Canada; ^2^ Department of Biochemistry, Microbiology and Immunology, University of Ottawa, Ottawa, Canada; ^3^ “John Fulcher” Neuro-oncology Lab, Imperial College, London, UK; ^4^ Centre for Cancer Therapeutics, Ottawa Hospital Research Institute, Ottawa, Canada; ^5^ Department of Pediatrics, University of Ottawa, Ottawa, Canada

**Keywords:** Glioblastoma multiforme, Bcl-xL, PDCD4, ABT-737, translation initiation, IRES

## Abstract

Glioblastoma multiforme (GBM) is the most common and aggressive form of tumor of the central nervous system. Despite significant efforts to improve treatments, patient survival rarely exceeds 18 months largely due to the highly chemoresistant nature of these tumors. Importantly, misregulation of the apoptotic machinery plays a key role in the development of drug resistance. We previously demonstrated that Bcl-xL, an important anti-apoptotic protein, is regulated at the level of translation by the tumor suppressor programmed cell death 4 (PDCD4). We report here a strong correlation between low expression of PDCD4 and high expression of Bcl-xL in adult *de novo* GBM, GBM tumor initiating cells, and established GBM cell lines. Importantly, high Bcl-xL expression correlated significantly with poor progression and patient survival. We demonstrate that re-expression of PDCD4 in GBM cells down-regulated Bcl-xL expression and decreased cell viability. Finally, we show that direct inhibition of Bcl-xL by small molecule antagonist ABT-737 sensitizes GBM cells to doxorubicin. Our results identify Bcl-xL as a novel marker of GBM chemoresistance and advocate for the combined use of Bcl-xL antagonists and existing chemotherapeutics as a treatment option for this aggressive tumor.

## INTRODUCTION

Glioblastoma multiforme (GBM) is the most common primary intrinsic tumor of the central nervous system in adults and one of the most aggressive types of cancer [1, 2]. Despite significant efforts to improve the outcome of patients with GBM, their median survival remains approximately 18 months, with a five-year survival of 5%. Both extensive infiltration of the adjacent brain that prevents radical surgical removal, and resistance to chemotherapy are the key factors of poor outcome. About 90% of GBM occurs *de novo*, while the remaining 10% result from the progression of a lower grade astrocytoma. Defined as secondary GBMs, these latter tumors show molecular and genetic changes that are different from *de novo* GBMs, including frequent mutation of p53 and mutation of IDH1[1, 2].

Low expression levels of the tumor suppressor programmed cell death 4 (PDCD4) have been correlated with poor outcome in patients with GBM. The frequent loss of PDCD4 in GBM is partly due to epigenetic silencing secondary to 5'CpG island methylation [3] as well as over-expression of microRNA 21 (miR-21) which targets PDCD4 mRNA for degradation [4]. Furthermore, frequent over-activation of kinases, in particular S6K1 and S6K2, observed in GBM leads to phosphorylation and subsequent degradation of PDCD4 [5-7]. PDCD4 plays key roles in a number of cellular processes including cell growth and invasion via inhibition of the AP-1 transcription factor as well as translation suppression through the eukaryotic initiation factor (eIF) 4A (reviewed in [8]). Since eIF4A is thought to be required for translation of virtually all cellular mRNAs, the PDCD4-dependent inhibition of eIF4A results in a decrease in global translation. Recently, however, several reports identified specific targets of PDCD4, thus pointing towards a novel role for this molecule in regulating selective translation of distinct mRNAs and not as a general inhibitor of translation [6, 9, 10]. Among the specific PDCD4 targets we identified the Bcl-2 family member Bcl-xL. Bcl-xL is an inhibitor of mitochondrial outer membrane permeabilization thus is a strong anti-apoptotic protein [6]. In addition it plays a role in p53 signaling [11] and cell cycle progression and checkpoints [12]. We demonstrated that PDCD4 specifically binds to and represses translation from the internal ribosome entry site (IRES) of Bcl-xL and that loss of PDCD4 removes the repression on the Bcl-xL IRES and results in an increase in Bcl-xL protein levels [6].

Given the known roles of Bcl-xL in regulation of apoptosis and chemoresistance, we sought to determine if the loss of PDCD4 expression observed in GBM causes elevated Bcl-xL expression, which could explain the high chemoresistance of GBM cells. Indeed, we find that low levels of PDCD4 correlate with high levels of Bcl-xL in both *de novo* GBM patient tumors and in established GBM cell lines and that high Bcl-xL correlates with poor progression and patient survival. Furthermore, we demonstrate that re-expression of PDCD4 in GBM cells results in a repression of Bcl-xL protein expression and a decrease in cell viability. Finally, we demonstrate that direct inhibition of Bcl-xL by the small molecule inhibitor ABT-737 results in a sensitization of GBM cells to doxorubicin. Our data identify Bcl-xL as a target of PDCD4 whose elevated levels contribute to high chemoresistance in GBM, thus providing a novel treatment option for this aggressive tumor.

### RESULTS

#### Loss of PDCD4 correlates with increased Bcl-xL in human GBM samples

Our previous work demonstrated the role of PDCD4 in regulating Bcl-xL, an inhibitor of apoptosis, through its IRES-mediated translation. Under normal proliferative conditions, we demonstrated that PDCD4 specifically and directly binds to and inhibits Bcl-xL IRES translation. However, when expression of PDCD4 is down-regulated, the repression on Bcl-xL is relieved thus resulting in an increase in Bcl-xL protein levels. These findings prompted us to investigate the link between PDCD4 and Bcl-xL in GBM.

**Figure 1:**
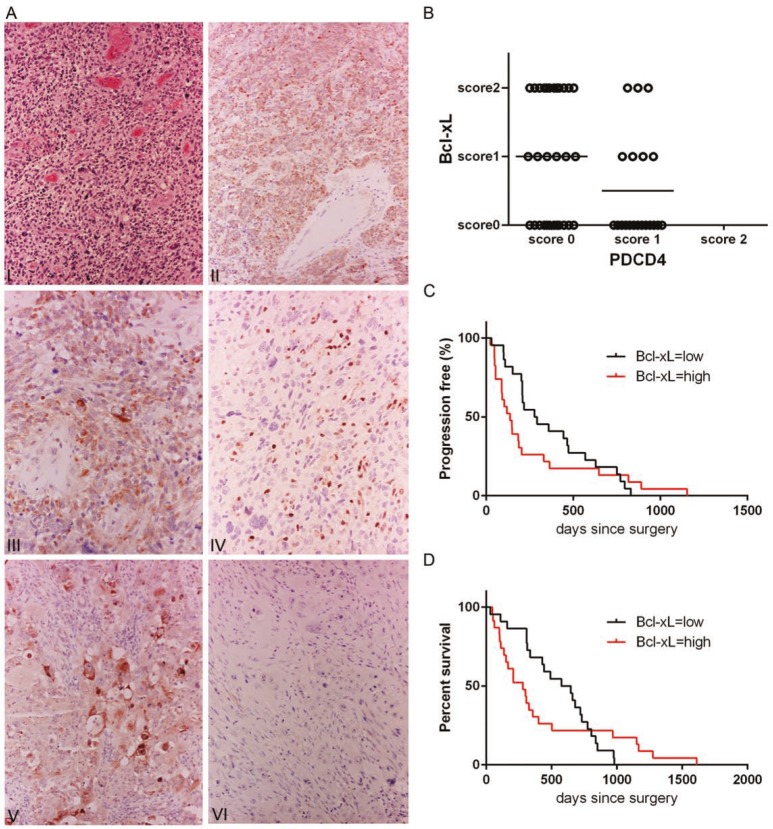
Patient GBM tumors show a correlation between low PDCD4 and high Bcl-xL levels (A) I – Hematoxylin-eosin stain of a representative case of glioblastoma multiforme (x10); II – diffuse Bcl-xL expression in neoplastic cells (immunoperoxidase x10); III – Granular cytoplasmic immunolabeling showing mitochondrial localization of Bcl-xL (immunoperoxidase x20); IV – PDCD4 is positive in endothelial cell and intratumoral lymphocytes (immunoperoxidase x20); V – Colorectal carcinoma as positive control for Bcl-xL (immunoperoxidase x20); VI – immunoreactions with omission of the primary antibody, (immunoperoxidase x20). (B) Dot plot representation of 50 cases of adult *de novo* GBM with respect to PDCD4 and Bcl-xL expression (Score 0 – expression between 0-5% was considered negative, Score 1 – expression between 5-50%, Score 2 – expression over 50%). More than 70% of Bcl-xL positive tumors are PDCD4 negative (Chi square test for trend, p=0.0469). (C) Kaplan-Meier curve showing a significant correlation between high Bcl-xL expression (score 1, 2) and progression (p=0.0187, Geham-Breslow-Wilcoxon test) and (D) significant correlation between high Bcl-xL expression (score 1, 2) and poor survival (p=0.0476, Geham-Breslow-Wilcoxon test).

In order to study the relationship between Bcl-xL and PDCD4 expression in a clinical setting, we investigated with immunohistochemistry a cohort of 50 human *de novo* GBMs. Twenty-six GBMs were positive for Bcl-xL, where 15 of them showed expression in more than 50% of neoplastic cells (score 2) (Figure 1A, 1B) and 11 showed focal expression (score 1). Thirty cases did not show any detectable PDCD4. Interestingly, 18 cases with no PDCD4 showed Bcl-xL positive cells and 12 PDCD4 positive cases had no Bcl-xL. Immunopositivity for Bcl-xL was cytoplasmic and granular in keeping with its mitochondrial localization (Figure 1A). Bcl-xL immunolabelling was also found in reactive astrocytes, a few microglial cells and some neurons. Sixteen tumors showed PDCD4 nuclear and/or cytoplasmic expression but it was only limited to a minority of tumor cells (score 1). In all 50 lesions, PDCD4 was present in endothelial and inflammatory cells, including perinecrotic macrophages (Figure 1A). Six out of the seven recurrent cases demonstrated diffuse Bcl-xL expression that was more intense than in the primary tumor. No change in PDCD4 was seen in recurrent tumors compared to the primary lesion. Chi square test for trend (p=0.0469) of this cohort suggested that PDCD4 likely regulates Bcl-xL protein in human samples, therefore confirming our previous *in vitro* data. The distribution of Bcl-xL and PDCD4 positive cases is represented in Figure 1B.

To understand if Bcl-xL and PDCD4 have any impact on patient outcomes, we correlated their expression with progression and survival (Figure 1C, 1D). Patients with Bcl-xL positive GBM (score 1 and 2) showed a median progression of 136 days and median survival of 280 days against median progression of 284.5 days and survival of 611 days of patients with Bcl-xL negative lesions. Gehan-Breslow-Wilcoxon test showed a significant difference between the Bcl-xL positive and Bcl-xL negative cases for both progression (p=0.0187) and survival (p=0.0476). The effect on MGMT status did not correlate with either progression or survival suggesting that Bcl-xL represents an important biomarker of chemoresistance of GBM.

**Figure 2:**
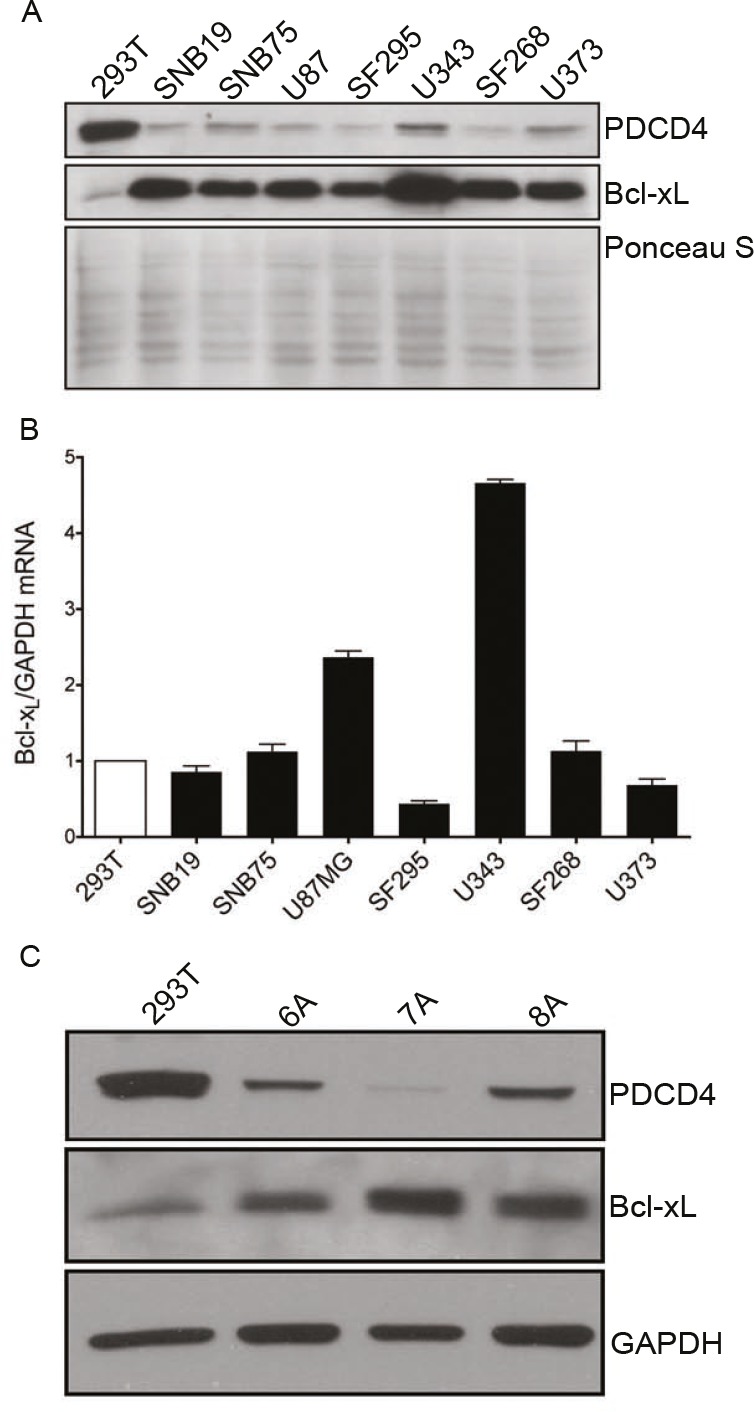
GBM cell lines and tumor initiating cells have low levels of PDCD4 and high levels of Bcl-xL protein (A) Western blot analysis of a panel of GBM cell lines indicating the correlation between low PDCD4 levels and high Bcl-xL levels. (B) qPCR analysis of GBM cell lines showing no increase in Bcl-xL versus GAPDH mRNA as compared to HEK293 reference. (C) Western blot analysis of GBM tumor initiating cells (TICs) showing correlation between low PDCD4 and high Bcl-xL levels.

In order to further explore the link between PDCD4 and Bcl-xL we examined a panel of established GBM cells lines and patient-derived tumor initiating cells (TICs). We measured the relative ratio of Bcl-xL and PDCD4 using HEK293 cells as a reference. In concordance with patient samples, we find that GBM cells exhibit low levels of PDCD4, which correlate with a robust expression of the Bcl-xL protein (Figure 2A, 2C). We have previously described the mechanism by which PDCD4 regulates expression of Bcl-xL through translational repression of the Bcl-xL IRES [6]. In accordance with this, other than U343 cells, we find that the levels of Bcl-xL mRNA do not differ significantly between HEK293 and GBM cells (Figure 2B), lending further support to the notion that PDCD4 is an inhibitor of Bcl-xL translation and that the loss of PDCD4 in GBM results in de-repression of Bcl-xL translation.

**Figure 3:**
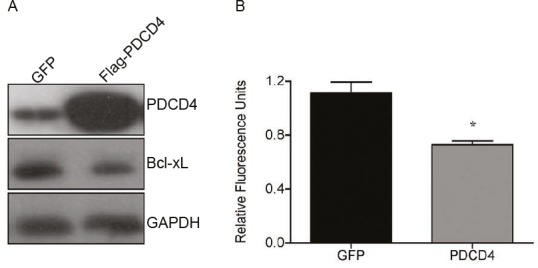
Re-introduction of PDCD4 into GBM causes reduction in Bcl-xL and cell viability (A) PDCD4 was overexpressed in U373 for 24 h followed by western blot analysis showing decrease in Bcl-xL. (B) Alamar blue analysis of cells overexpressing PDCD4 for 24 h to measure cell viability (* p<0.05).

To further demonstrate the causal link between PDCD4 and Bcl-xL expression, U373 cells were transiently transfected with GFP- or PDCD4-expressing plasmids and the expression of Bcl-xL was determined by Western blot analysis. We find that restoring the expression of PDCD4 resulted in a significant decrease in Bcl-xL (Figure 3A). Furthermore, since Bcl-xL is a known inhibitor of apoptosis, we were interested in determining the effect that the PDCD4-mediated decrease in Bcl-xL expression would have on cell viability. We thus measured cell viability in GFP- or PDCD4-transfected cells by Alamar blue analysis. We observed that restoring PDCD4 levels in U373 cells resulted in a significant reduction in cell viability (Figure 3B). Taken together, this data suggests that the apoptotic resistance typically observed in GBM cells is due in part by the large increase in the expression of Bcl-xL, which is a result of the loss of PDCD4. More importantly, our data suggest that by manipulating the levels of Bcl-xL, GBM cells could be sensitized to chemotherapeutics.

#### Inhibition of Bcl-xL sensitizes GBM cells to Doxorubicin

**Figure 4:**
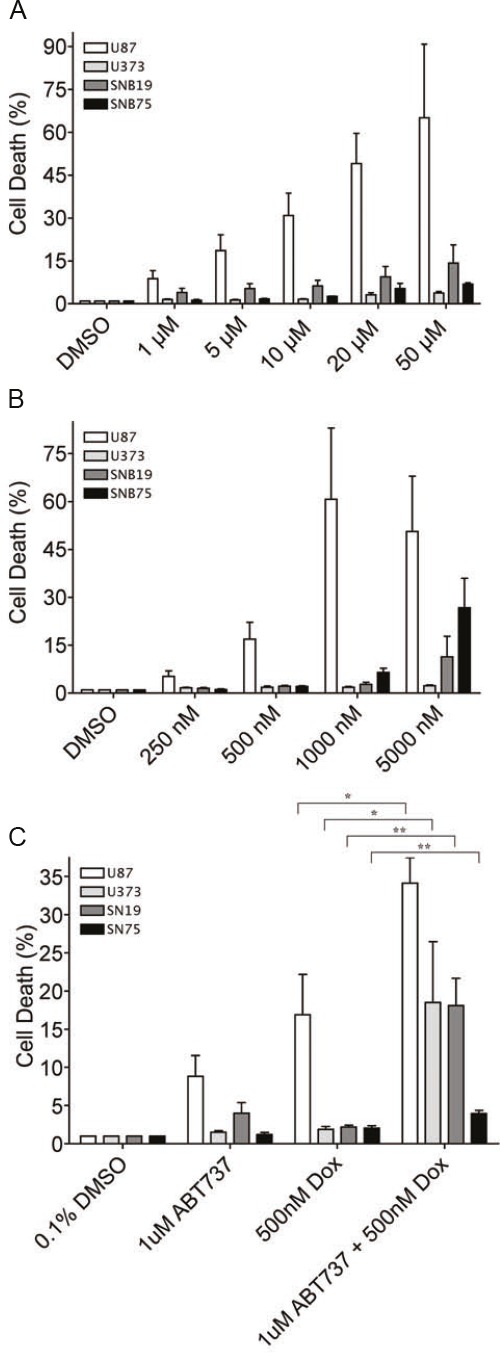
ABT-737 sensitizes GBM cells to doxorubicin (A) Cytotoxicity was measured by incorporation of Yoyo-1 dye after treatment of indicated cell lines with increasing concentrations of doxorubicin for 24 h. (B) Cytotoxicity was measured after treating indicated cell lines with increasing concentrations of ABT-737 in the presence of Yoyo-1. (C) Cytotoxicity was measured after treating indicated cells with ABT-737 (1uM), doxorubicin (dox, 500nM), or a combination of both in the presence of Yoyo-1 (* p<0.05, ** p<0.01).

Since re-introduction of PDCD4 protein into tumor cells is likely not a feasible therapeutic option, we sought to explore if targeting downstream of PDCD4 would offer an effective strategy. We therefore chose to inhibit Bcl-xL using the small molecule inhibitor ABT-737 in combination with the chemotherapeutic drug doxorubicin. We expected that inhibition of Bcl-xL would sensitize cells to doxorubicin since high Bcl-xL levels would no longer inhibit the apoptotic machinery. Four different GBM cell lines (U87, U373, SNB19, SNB75) were treated with ABT-737 alone or in combination with doxorubicin for 24 hours and cell viability was determined (Figure 4). We observed that treatment of three out of four GBM cell lines with ABT-737 alone resulted in minimal cell death even at highest concentrations of the inhibitor (Figure 4A). In contrast, one cell line, U87, was sensitive to ABT-737 at high concentrations. Similarly, with the exception of U87 cells, the treatment of these cells with doxorubicin alone resulted in minimal cell death (Figure 4B). However, combined treatment with sublethal doses of doxorubicin (500nM) and ABT-737 (1 uM) significantly increased cytotoxicity in all four cell lines tested (Figure 4C).

### DISCUSSION

Despite the recent advances in surgical resection techniques, radiotherapy planning and dose delivery, and stratified targeted medical treatment, more than 90% of patients with GBM succumb to disease within 18 months from onset and show an overall median progression-free survival time of approximately 7 months [2]. Recent clinical trials have achieved better outcomes with median overall survival of up to 22 months but such an improvement is likely influenced by better supportive care, more aggressive salvage therapy and a more stringent selection of patients enrolled. In fact, good preoperative and postoperative performance status and extensive neurosurgical tumor resection are important factors influencing the outcome. Extensive infiltration of normal brain, intra and inter-tumoral molecular and genetic heterogeneity, and chemoresistance of neoplastic cells are the key determinants of poor treatment response.

Chemotherapy represents the mainstream treatment modality in GBM but glioma cells characteristically show simultaneous resistance towards cytotoxic drugs. Such multidrug resistance can occur at the beginning of treatment or be acquired during chemotherapy. The mechanisms causing chemoresistance are complex and multifactorial [13]. In addition, defects in the apoptotic pathway are also important in tumor drug resistance, which can be secondary to either over-activation of anti-apoptotic proteins or to the loss of expression or loss of function of pro-apoptotic molecules [14]. In our previous studies, we demonstrated that the tumor suppressor protein PDCD4 plays a critical role in regulating IRES mediated translation of key anti-apoptotic factors including Bcl-xL [6]. We showed that under normal conditions, PDCD4 directly binds to and inhibits translation of Bcl-xL mRNA. However, upon loss of PDCD4, the repression is relieved thus resulting in IRES mediated translation and an increase in protein production. High levels of Bcl-xL have been associated with chemoresistance by inhibiting the cell from undergoing apoptosis upon the addition of stressors [15]. This chemoresistance is through Bcl-xL's ability to prevent mitochondrial outer membrane permeabilization and cytochrome c release. Since PDCD4 is a known tumor suppressor, and is lost in a variety of tumors including GBM, we were interested in determining if this loss also corresponds with an increase in Bcl-xL, which would contribute to the highly chemoresistant nature of GBM tumors. Indeed, we identified a correlation between low levels of PDCD4 and high levels of Bcl-xL in a cohort of 50 primary GBM tumors. Furthermore, we observed a significant correlation between Bcl-xL and tumor progression and patient survival. This data suggests that Bcl-xL may be a good prognostic tool to identify patient outcome and response to chemotherapy, independent of MGMT status. Our results differ from the study published by Cartron et al [16] where the authors examined 13 members of the Bcl-2 family, including Bcl-xL, and found no correlation with overall survival. Their analysis was based on Western blot and Elisa data and the results were evaluated semi-quantitatively attributing score 0 to tumors with low expression and 1 to tumors showing high expression. Given the heterogeneity of the microenvironment of GBMs that contains several non-neoplastic cell populations [17], we decided to assess Bcl-xL by immunoperoxidase immunohistochemisty on tissue sections. This allowed us to count the expression in tumor cells only. With this approach entrapped neurons, microglia and reactive astrocytes that were seen to express the protein were excluded from our assessment. In contrast, expression of Bcl-xL in non-neoplastic cells may have affected Cartron et al. results that were obtained using tumor homogenates. Given the retrospective nature of our study, semi-quantitative analysis of Bcl-xL was based on number of positive cells rather than intensity of expression accounting for another difference between the two studies. Correlation with outcome was obtained considering positive cases with those with more than 5% of expression irrespective of intensity. With a similar semi-quantitative approach Yoshimine et al [18] observed a correlation between lower survival and increased expression of Bcl-xL in urothelial carcinoma, further suggesting a role for this molecule in cancer progression.

Furthermore, we also observed this correlation in tumor initiating cells and in a panel of established GBM cell lines, which we utilized for further analysis. Importantly, we did not see a significant change in Bcl-xL mRNA levels that would account for the drastic increase in protein levels. This finding suggests that transcription and/or stability of the mRNA is not a contributing factor; however, translation of the Bcl-xL mRNA is critical for the increase in protein. This further supports our previous findings that PDCD4 can negatively regulate Bcl-xL IRES mediated translation. To verify that PDCD4 directly affects Bcl-xL in GBM cells, we rescued PDCD4 expression, which resulted in reduction in Bcl-xL levels and importantly, a reduction in cell viability. In order to use this therapeutically to our advantage, we decided to target downstream of PDCD4 since overexpressing a protein in patient tumor samples is likely not a feasible option. We treated four different GBM cell lines with the chemotherapeutic doxorubicin, which resulted in minimal cell death at low concentrations likely due to the high levels of Bcl-xL. Increase in cell death was observed at high concentrations of the drug in accordance with previous reports [19]. Importantly, by directly inhibiting Bcl-xL with the chemical inhibitor ABT-737, we were able to sensitize these cells to the effects of low concentrations of doxorubicin. These data clearly indicate the importance of monitoring the levels of Bcl-xL in patient tumors as well as the possibility of using ABT-737 as an adjuvant to existing chemotherapeutics in treating GBM tumors.

## MATERIALS AND METHODS

### Patient selection

From the Brain Tumor Registry at Imperial College, London UK, we have selected 50 adult patients operated *de novo* GBM whom the clinical history, pre-operative and post-operative MRI scan, and data on post-operative radio-chemotherapy and follow-up were available for review (Supplementary Table) and examined their tumors for the expression of PDCD4 and Bcl-xL. Given the heterogeneous nature of GBM, we chose samples obtained from maximally safe surgical debulking rather than stereotactic biopsies because they allowed us to examine as much tumor tissue as possible and investigate the full extent and distribution of the two proteins. We chose supratentorial, hemispheric GBMs because gross total excision is not achievable in tumors occurring in the subcortical grey matter, cerebellum, brainstem and spinal cord. None of the patients had previous evidence of a lower grade astrocytoma. In order to correlate PDCD4 and Bcl-xL expression with patients' outcome, we selected patients that had Karnofsky's performance status at onset of 70 or higher. Evidence of disease progression documented at follow-up neuroimaging and survival time were used as measures of outcome and were calculated in days from the day of operation.

Of the 50 patients, 19 were female; the mean age at the time was 57 years and 2 months (range between 31yrs 5mo– 78yrs 7mo; median 58yrs). Progression time ranged between 23-1154 days (average 316 days; median 207 days). Survival ranged 30-1611 days – average 492 days; median 355 days). Five patients were alive at the time of the last follow-up and four of them were clinically stable with no evidence of progression at neuroimaging. Ten patients had a second operation following progression after chemoradiation but diagnostic tissue for further immunostains was only available in 7 cases. Two of these 10 patients had progression after the second operation. All patients had consented for their tissue to be used for research.

### Pathological assessment

Tissue was routinely fixed in formalin and embedded in paraffin (FFPE). The original HE-stained sections of each case were re-examined to confirm the diagnosis and the most representative sample from each case was used for immunohistochemical stains. Five micron sections were cut from each block for immunohistochemistry. All lesions were routinely assessed for *MGMT* gene promoter methylation and expression of the mutant IDH1^R132H^. Peroxidase immunohistochemitry was performed on FFPE tissue sections with antibodies directed against PDCD4 (Rabbit polyclonal, Rockland at the dilution of 1:350 following antigen retrieval 20 in steamer in citrate buffer at pH 6) and anti-Bcl-xL (Rabbit polyclonal Cell Signalling at the dilution of 1:200, following antigen retrieval 20 in steamer in citrate buffer at pH 6). Briefly, sections were dewaxed in xylene and dehydrated in decreasing alcohols to distilled water. Endogenous peroxidase quenching was obtained by incubating the sections in 0.3% hydrogen peroxide for 30 min. Immunostains were then performed with an automated immunostainer (Leica bond III, Leica Microsystems Ltd, Milton Keynes, UK). Sections were counterstained in Harris' hematoxylin, dehydrated in progressive alcohols and xylene and coverslipped. Sections of pharyngeal tonsils were used as a positive control for PDCD4 and sections of colon cancer were used as positive control for Bcl-xL. Immunostains following similar protocol but with omission of the primary antibody were performed as negative controls. The extent of expression of the two proteins was assessed semi-quantitatively on tissue sections. Expression between 0-5% was considered negative (score 0), while tumors featuring more than 50% of positive cells were scored 2. Any tumor showing between 5 and 50% positivity was scored 1.

### Cell Culture

Human embryonic kidney (HEK293), and human glioblastoma (SNB19, SNB75, U87, SF295, U343, SF268, U373) cells were maintained in standard conditions in Dulbecco's modified Eagle's medium supplemented with heat-inactivated 10% fetal calf serum, 2 mM L-glutamine, and 1% antibiotics (100 units/ml penicillin-streptomycin). Cells were transfected using Fugene (Roche) as per the manufacturer's protocol. For the isolation of tumor initiating cells, consent was obtained from patients in accordance with a protocol approved by the Ottawa Hospital Research Ethics Board. Surgical samples were digested with Accutase (Sigma-Aldrich, Oakville, ON, Canada), filtered through 100 μM and 40 μM nylon mesh filters, and plated on laminin-coated plates in Neurobasal A medium supplemented with B27, N2 (all from Life Technologies, Burlington, ON, Canada), EGF and FGF (Peprotech, Rocky Hill, NJ, USA) as described by Pollard et al [20]. Cells were cultured in 5% O_2_/CO_2_ at 37°C. Debris and other cell types were removed by media changes once tumor initiating cells had adhered. Cells were characterized for chromosomal abnormalities typical of glioblastoma using chromogenic *in situ* hybridization, positive expression of nestin by immunofluorescence, the ability to form neurospheres in the absence of laminin, the ability to differentiate in response to serum exposure and/or growth factor withdrawal, and the ability to form invasive glioblastoma in immunocompromised mice as described by Gont et al [21].

### Western blot

Cells were lysed in RIPA buffer for 15 min on ice followed by centrifugation at 12,000 × g for 15 min to pellet cell debris. Protein concentrations were measured by a Bradford Assay (Bio-Rad) and equal amounts of proteins were separated by SDS-PAGE and transferred to a PVDF membrane. Western blotting was performed using rabbit anti-PDCD4 (Rockland), rabbit anti-Bcl-xL (Cell Signaling Technology), or mouse anti-GAPDH (Advanced ImmunoChemical Inc), followed by species-specific horseradish peroxidase-conjugated secondary antibodies (Cell Signaling Technology). Antibody complexes were detected using ECL (GE Biosciences) and X-ray film (Kodak).

### RNA extraction, qRT-PCR

Total RNA was isolated using the Absolutely RNA Miniprep kit (Stratagene). Reverse transcription was done using the First-strand cDNA synthesis kit (GE Biosciences) and quantitative PCR was performed using the QuantiTect SYBR green PCR kit (Qiagen) with gene specific primers for Bcl-xL (QuantiTect Primer Assay; Qiagen) and GAPDH [6].

### Alamar Blue Analysis

U373 cells were transfected with GFP or GFP-PDCD4 for 24 h followed by analysis of cell viability by Alamar Blue (Invitrogen) as per manufacturer's protocol.

### Cytotoxicity Assays

Cells were seeded in a 96-well plate at 5000 cells/well for 24 h. Cells were treated with ABT-737, Doxorubicin, or their combinations for 24 h in the presence of Yoyo-1 (Molecular Probes) and the percentage of Yoyo-1 positive cells versus total cells as indicated by Vybrant DyeCycle Green (Molecular Probes) was measured using the IncuCyte Live-Cell Imaging System (Essen Bioscience).

### Statistical analysis

With the exception of patient data all data are expressed as means +/− standard deviation (s.d), with a minimum of three independent experimental replicates unless otherwise noted. Chi square test for trend, Geham-Breslow-Wilcoxon test, and student's t-test were performed to determine data significance using GraphPad Prism version 5.04 for Windows (GraphPad Software, San Diego, CA).

## Supplementary Tables


