# Supplementary material for: Loss of PDCD4 contributes to enhanced chemoresistance in Glioblastoma Multiforme through de-repression of Bcl-xL translation
Source: Oncotarget. 2013 Jul 28;4(9):1365–72. doi: 10.18632/oncotarget.1154 (PMC3824522; doi:10.18632/oncotarget.1154)
Supplement: Supplementary file 1 [file oncotarget-04-1365-s001.pdf]

Supplementary Table: Clinical history, pre-operative and post-operative MRI scan, and data on post-operative radio-chemotherapy and follow-up of 50 adult GBM used in this study.

ID	gender	age in months	date of surgery	Pdcd4	Bcl-xT	MGTM MSP	mIDH1	CD133	time of progression	days progression	treatment		date of death	days survival	other operations	Pdcd4	Bcl-xT
1	M	432	27.02.2001	0	2	um	positive	a few cells	19.07.2001	148	TEMO 75 + RT	Dead	28.07.2001	151			
2	M	378	15.05.2001	0	0	u	negative	a few cells	23.08.2002	465	TEMO + RT; temo + Irinotecan	Dead	11.12.2002	575			
3	M	600	16.05.2001	0	1	um	negative	negative	01.02.2002	330	TEMO + RT - cisplatin - doxorubicin	Dead	18.06.2002	399			
4	M	905	18.07.2001	0	2	um	negative	negative	01.12.2001	136	NONE	Dead	01.12.2001	136			
5	M	692	30.11.2001	1	0	u	negative	negative	01.05.2002	152	TEMO + RT; Irinotecan	Dead	01.11.2002	336			
7	M	724	12.05.2003	1	1	um	negatve	negative	17.02.2005	647	TEMO 75 + RT	Dead	08.11.2006	1276	second surgery - 08/02/2005		
8	M	750	28.07.2003	0	0	u	negative	a few cells	01.03.2004	217	TEMO 75 + RT	Dead	17.05.2005	659			
9	F	601	10.09.2003	0	1	u	positive	a few cells	01.04.2004	204	TEMO 75 + RT; FAFEC	Dead	12.07.2004	306			
10	F	797	12.03.2004	1	0	u	negative	negative	01.10.2005	568	TEMO 75 + RT; Adjuvant TEMO	Dead	19.12.2005	647			
11	F	736	25.08.2004	0	2	um	negative	negative	01.02.2007	890	TEMO 75 + RT; Adjuvant TEMO; PCV; TEMO	Dead	19.10.2007	1150			
12	M	629	15.07.2005	0	0	um	negative	negative	01.10.2006	443	TEMO 75 + RT; Adjuvant TEMO	Dead	25.05.2007	679			
13	M	566	28.11.2005	1	2	um	negative	negative	stable - 76 months	stable	TEMO 75 + RT; Adjuvant TEMO	Alive	///	2652			
14	M	945	23.12.2005	0	2	um	negative	negative	28.06.2006	187	TEMO 75 + RT	Dead	18.10.2006	299			
15	M	639	12.01.2006	0	0	um	negative	negative	21.04.2008	830	TEMO 75 + RT	Dead	12.05.2008	851			
16	F	688	08.02.2006	1	0	um	negative	negative	01.11.2007	358	TEMO 75 + RT; Adjuvant TEMO; Avastin	Dead	22.04.2008	804			
17	F	641	22.02.2006	0	0	um	negative	negative	01.06.2006	99	TEMO 75+ RT; 1 cycle PCV	Dead	01.01.2007	313			
18	M	665	13.03.2006	0	0	um	negative	occasional	01.10.2006	202	TEMO 75 + RT; 2 cycles PCV	Dead	17.07.2007	491			
19	M	627	17.05.2004	0	0	um	negative	negative	01.06.2006	795	TEMO 75 + RT; Adjuvant TEMO; PCV	Dead	16.12.2006	975	second surgery - 26.06.2006	1	2 (strong)
20	F	821	09.07.2006	0	1	um	negative	negative	29.08.2006	47	NONE	Dead	29.08.2006	51			
21	F	714	02.01.2007	0	2	um	negative	negative	01.04.2007	89	TEMO 75 + RT	Dead	24.11.2007	326	second surgery - 08/05/2007	0	2
25	M	640	15.02.2007	0	0	um	negative	negative	01.06.2008	472	TEMO 75 + RT; Adjuvant TEMO; PCV	Dead	16.02.2009	732	second surgery 05/08/2008	0	2
26	M	805	02.03.2007	0	1	u	negative	negative	27.07.2007	147	TEMO 75 + RT	Dead	24.09.2007	206			
27	F	636	13.04.2007	1	2	um	negative	negative	06.05.2007	23	TEMO 75 + RT	Dead	21.07.2007	99			
28	M	860	04.06.2007	0	1	um	negative	negative	01.09.2007	89	TEMO 75 + RT	Dead	14.09.2007	102			
29	F	484	05.09.2007	1	0	um	negative	negative	23.12.2007	109	TEMO 75 + RT	Dead	23.12.2007	109			
30	M	778	21.12.2007	0	0	um	negative	negative	15.07.2008	207	TEMO 75 + RT; Adjuvant TEMO	Dead	22.02.2009	429			
32	F	755	20.02.2008	0	0	um	negative	occasional	15.09.2008	208	TEMO 75 + RT; Adjuvant TEMO	Dead	25.12.2008	309			
34	M	811	07.03.2008	0	2	um	negative	negative	24.04.2008	48	NONE	Dead	24.06.2008	280			
35	M	553	19.04.2008	1	0	um	negative	negative	05.02.2009	292	TEMO 75 + RT; Adjuvant TEMO; Avastin	Dead	06.07.2009	443			
37	M	668	08.05.2008	1	1	um	negative	a few cells	01.07.2008	54	TEMO 75 + RT	Dead	21.10.2008	166			
38	M	917	22.05.2008	0	2	um	negative	a few cells	17.06.2008	47	NONE	Dead	17.06.2008	47			
39	M	696	04.03.2010	0	0	um	negative	negative	12.06.2010	100	TEMO 75 + RT	Dead	12/08/2010	161			
40	F	732	24.07.2008	0	2	um	negative	negative	21.09.2011	1154	TEMO 75 + RT; Adjuvant TEMO	Dead	21/12/2012	1611	second surgery - 07/06/2011	0	2
41	M	468	28.08.2008	1	0	um	negative	a few cells	07.09.09 & 16.08.2012	375 and 1149	TEMO + RT #6; PCV #4	Alive	///	1648	second surgery - 16/03/2009	0	2 (strong)
42	F	733	04.09.2008	1	0	um	negative	negative	22.09.2010 & 24.03.2011	750 and 931	TEMO 75 + RT; Adjuvant TEMO; Avastin	Dead	10/05/2011	979			
43	F	739	19.09.2008	0	1	um	negative	negative	15.01.2009	118	TEMO 75 + RT; PCV	Dead	10.05.2011	968			
44	F	776	03.10.2008	1	0	um	negative	negative	23/06/2010	628	TEMO 75 + RT; Adjuvant TEMO	Dead	16.11.2010	775			
45	M	528	30.10.2008	1	1	um	negative	some	stable - 11.09.2012	stable	TEMO + 75 + RT 6#; 6# Adjuvant TEMO 150/2[illegible]	Alive	///	1412			
47	M	755	09.12.2002	0	2	um	negative	negative	10.06.2003	183	Radical RT but no chemo	Dead	03.07.2003	206			
48	M	866	05.12.2008	0	2	u	negative	negative	19.03.2009	104	TEMO 75 + RT	Dead	25.03.2009	110			
49	M	733	12.12.2008	1	0	u	negative	negative	15.09.2009	277	TEMO+RT #6; Adjuvant TEMO 150/20	Dead	17.10.2009	309			
50	M	570	05.01.2009	1	2	um	negative	negative	16.04.2009	91	#6 TEMO + RT	Dead	26.12.2009	355	second surgery - 08/05/2009	not done	not done
51	F	461	04.04.2008	0	2	um	negative	a few cells	30/06/2010	817	TEMO + RT - second debulking 16/07/2010	Dead	15.06.2011	1167	second surgery - 16/07/2010	0	1
52	M	746	21.11.2008	1	0	um	negative	negative	21.12.2008	30	NONE	Dead	21.12.2008	30			
54	M	805	05.06.2009	1	1	um	negative	a few cells	03.06.2010	363	TEMO+RT #6 - no response to treatment	Dead	21/10/2010	503	second surgery - 07/07/2010	not done	not done
55	M	916	09.06.2009	1	1	um	negative	a few cells	01.08.2009	53	Palliative RT #6	Dead	10.08.2009	62			
57	F	571	24.07.2009	1	0	u	negative	a few cells	stable - 14/12/2012	stable	TEMO+RT #6	Alive	///	1239	second surgery - 11/11/2009	not done	not done
58	F	380	13.08.2009	0	2	u	negative	a few cells	stable - 20/11/2012	stable	Gliadel wafers - TEMO 75 + RT#6; Adjuvant	Alive	///	1195			
59	F	717	22.06.2004	0	0	u	negative	a few cells	01.08.2006	770	TMZ + RT and adjuvant TMZ 6 cycles	Dead	08/10/2006	838	second surgery - 19/09/2006	0	2 (strong)
61	F	678	08.05.2009	1	0	um	negative	occasional	01.12.2009	207	TMZ + RT	Dead	27/04/2011	719			
	gender	age in weeks	date of surgery	PDCD4	Bcl-xT	MGTM MSP	mIDH1	CD133	date of progression	days progression	treatment	survival	date of death	days survival	second surgery	PDCD4	Bcl-xT
